# The Lung in Primary Immunodeficiencies: New Concepts in Infection and Inflammation

**DOI:** 10.3389/fimmu.2018.01837

**Published:** 2018-08-08

**Authors:** Ulrich Baumann, John M. Routes, Pere Soler-Palacín, Stephen Jolles

**Affiliations:** ^1^Department of Paediatric Pulmonology, Allergy and Neonatology, Hannover Medical School, Hannover, Germany; ^2^Division of Asthma, Allergy and Clinical Immunology, Department of Pediatrics, Medical College of Wisconsin, Milwaukee, WI, United States; ^3^Pediatric Infectious Diseases and Immunodeficiencies Unit, Hospital Universitari Vall d’Hebron, Institut de Recerca Vall d’Hebron, Universitat Autònoma de Barcelona, Barcelona, Spain; ^4^Immunodeficiency Centre for Wales, University Hospital of Wales, Cardiff, United Kingdom

**Keywords:** primary immunodeficiency, lung complications, immunoglobulin, comorbidity, bronchiectasis, granulomatous-lymphocytic interstitial lung disease, pulmonary functional tests, lung computed tomographic scan

## Abstract

Immunoglobulin replacement therapy (IGRT) has contributed critically to the management of primary antibody deficiencies (PAD) and the decrease in pneumonia rate. However, despite adequate IGRT and improved prognosis, patients with PAD continue to experience recurrent respiratory tract infections, leading to bronchiectasis and continuing decline in lung function with a severe impact on their quality of life. Moreover, non-infectious inflammatory and interstitial lung complications, such as granulomatous-lymphocytic interstitial lung disease, contribute substantially to the overall morbidity of PAD. These conditions develop much more often than appreciated and represent a major therapeutic challenge. Therefore, a regular assessment of the structural and functional condition of the lung and the upper airways with appropriate treatment is required to minimize the deterioration of lung function. This work summarizes the knowledge on lung complications in PAD and discusses the currently available diagnostic tools and treatment options.

## Introduction

**Figure 1:**
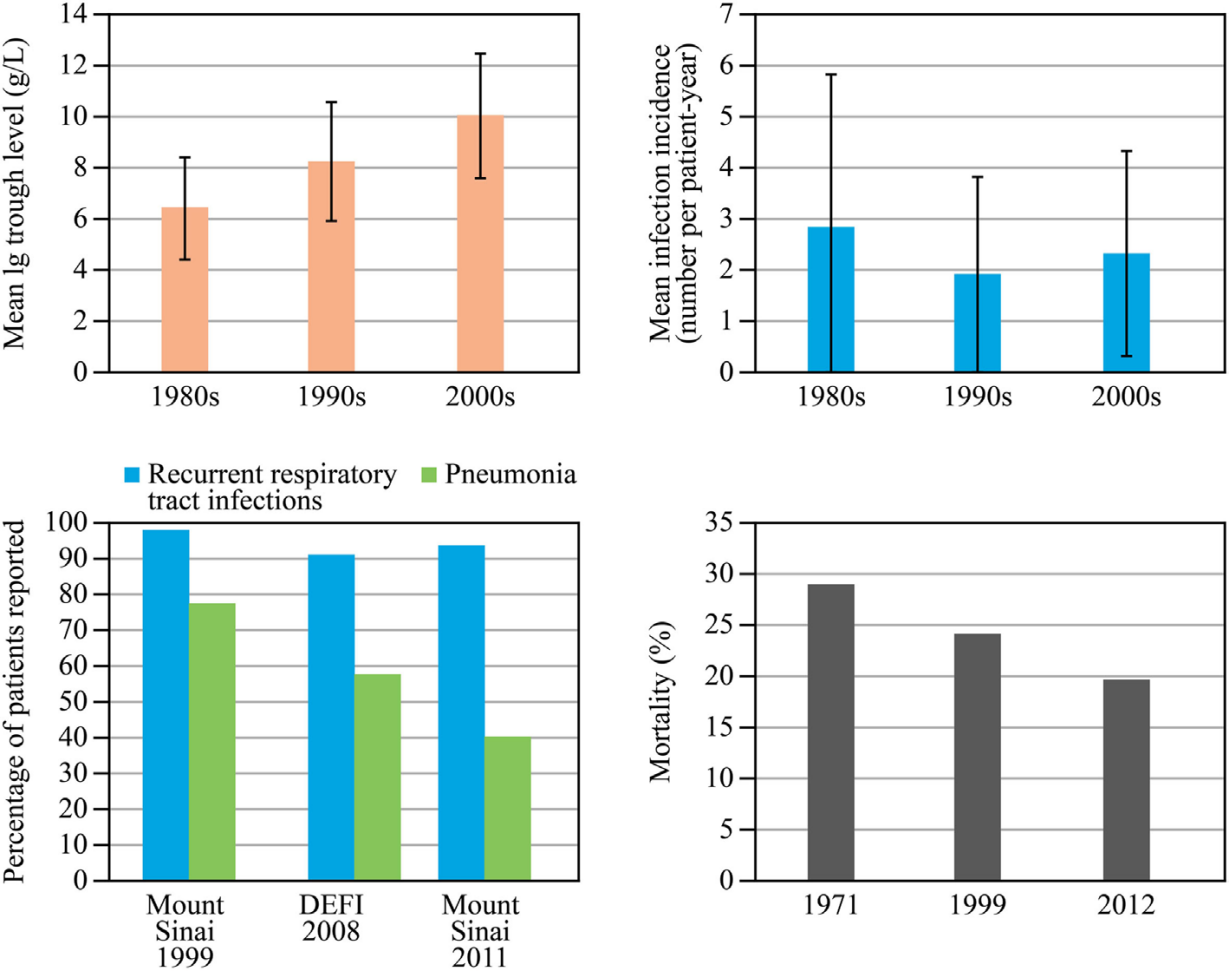
Development of immunoglobulin replacement therapy and infection rates since 1990s. Data for immunoglobulin (Ig) trough levels (top left) and infection rates (top right) are from a cohort of 115 patients with common variable immunodeficiency (CVID) or X-linked agammaglobulinemia reported by Lucas et al. (1). A combined analysis of data on the percentage of patients experiencing pneumonia and recurrent respiratory tract infections (bottom left) is shown in three large series of CVID patients in 1999, 2008, and 2011 (two from the same cohort in the United States 12 years apart, 248 and 252 patients, and one from a French cohort, 473 patients); reproduced with kind permission from 2014 British Society for Immunology, Clinical and Experimental Immunology, 178: 67–69 (2). Mortality data (bottom right) for 1971 are from Healy et al. (6); those for 1999 and 2012 are from a single cohort follow up reported by Resnick et al. (7). DEFI, a French national study. Abbreviation: PAD, primary antibody deficiencies.

Immunoglobulin (Ig) replacement therapy (IGRT) has made a critical difference in the treatment of primary antibody deficiencies (PAD). Improvements in care have led to a decrease in severe bacterial infections, notably pneumonia. Since the introduction of modern IGRT in the 1980s, Ig doses have increased, resulting in significantly higher plasma IgG trough levels (Figure 1) (1). The incidence of bacterial pneumonia in patients with PAD has been nearly halved (2) and the major risk factors—low IgA levels, combination of low IgA and IgM levels (3, 4), low IgG levels despite replacement IGRT, chronic sinusitis, bronchiectasis, and low number of class-switched memory B cells—are known, directing risk stratification, informing monitoring, and detecting early symptoms. Over a period of 40 years, the mortality rate in patients with common variable immunodeficiency (CVID) has decreased steadily from 29% in 1971 [before the introduction of intravenous IgG (IVIG)] to 24% in 1999 and 19.6% in 2012 (5–7). An analysis of the European Society for Immunodeficiencies (ESID) registry data provided an overall mortality estimate of 15%, with 75th and 60th percentiles for survival of 25 and 41 years after diagnosis [95% confidence intervals (CI) 22–33 and 33–41 years, respectively] (8).

However, despite improved therapy and prognosis, patients with PAD continue to experience non-infectious chronic lung disease and recurrent respiratory tract infections even with adequate IGRT (i.e., IGRT that raises trough IgG levels to within the range of healthy individuals), leading to an ongoing decline in lung function—in fact, the decline is greater than that in heavy smokers (9, 10). Pulmonary complications are common in a wide range of PAD and have a severe impact on patients’ quality of life (11–13). At the time of diagnosis, most patients have already suffered from recurrent bacterial pulmonary infections, leading to bronchiectasis and/or obstructive lung disease in some patients. Additionally, non-infectious complications are an increasing cause of morbidity and mortality in PAD. For example, structural and functional lung impairment is now recognized as an important risk factor for early mortality in CVID (7). In fact, these non-infectious complications—caused by immune dysregulation and not limited to the lung—have emerged as a major diagnostic and therapeutic challenge.

Therefore, active and accurate screening is mandatory even in “well-controlled” patients, but the absence of structured protocols for follow-up is a major challenge toward identifying and treating lung complications (14). This review focuses on the current challenges in the management of lung disease in patients with PAD.

## Immunity and Immunodeficiency of the Respiratory System

The lung is an organ of enormous surface area combined with delicate structure that encounters a large amount and variety of pathogens capable of causing infection. Igs play a major role in the protection of the lung against infection, with specific roles for different isotypes (15). Different Ig isotypes are dominant in different parts of the lung in the airways: the respiratory surface in the upper and lower airways is covered predominantly with secretory IgA (sIgA) and IgM, while in the alveolar space, IgG is the dominant isotype (16, 17).

Both sIgA and IgM at the bronchial surface mostly derive from the mucosa associated lymphatic tissue, rather than from the systemic circulation (15, 18, 19). sIgA prevents bacterial adhesion or neutralizes toxins without causing an inflammatory response, in contrast to IgG and monomeric IgA (20). IgM activates the complement system, which enhances opsonization of pathogens. Due to its multimeric structure, IgM is highly effective for agglutination, especially of viruses (20). Alveolar IgG originates from the systemic circulation by passive diffusion and effectively prevents bacterial infections such as pneumonia (19, 21).

In most patients with PAD, both systemic IgG and local IgA are absent or dysfunctional; indeed, one of the criteria in the definition of CVID in the revised ESID and International Consensus guidelines is low IgA and/or low IgM (22, 23). sIgA seems to play a negligible role for airway defense because individuals with selective IgA deficiency are commonly healthy. However, the observation that patients with CVID suffer from airway infections more severely with very low IgA levels (i.e., less than 0.07 g/L) compared with those with higher IgA levels (24) argues that IgA can compensate for IgG deficiency in airway defense, at least in part. Combined deficiency of IgA and IgM seems to be even more detrimental if IgG is lacking (25) suggesting that IgM is also a cofactor for airway defense.

Without IGRT, the most common infection sites in patients with X-linked agammaglobulinemia (XLA) and CVID are in the airways (2, 26, 27). The same types of bacteria reside in the upper and lower airways in PAD, with analogous evidence in the setting of cystic fibrosis (CF) for *Pseudomonas aeruginosa* (28). The concept that the upper airways form a gateway and a reservoir of infection for the lower airways was supported by the same genetic fingerprint bacteria collected from nasal and bronchial simultaneously (29). Therefore, the optimal pulmonary management of patients with PAD must encompass the upper airway, the “gateway to the lungs.”

## Respiratory Infections in PAD: What is Being Missed

**Figure 2:**
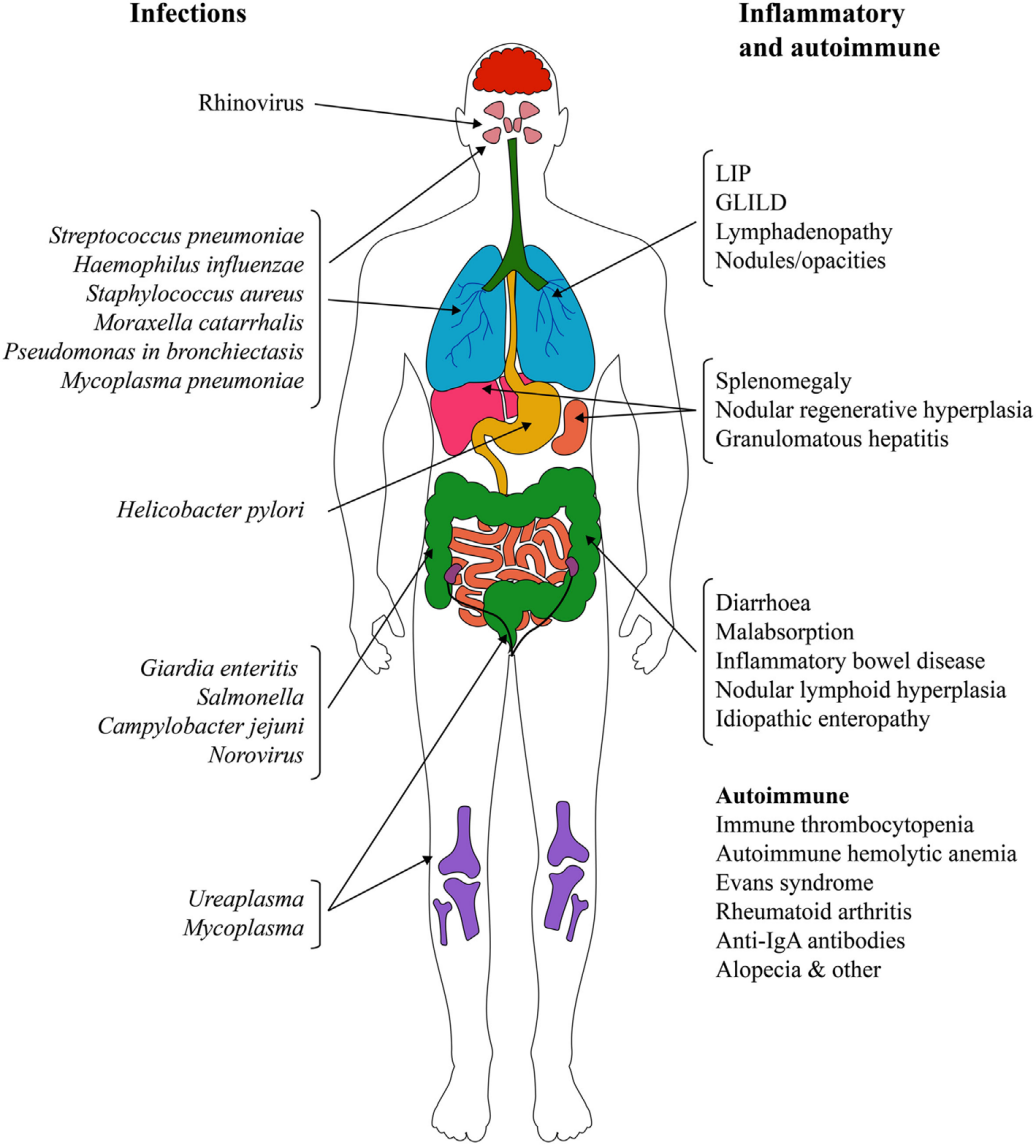
Infectious and non-infectious complications in primary antibody deficiencies. Abbreviations: GLILD, granulomatous-lymphocytic interstitial lung disease; IgA, immunoglobulin A; LIP, lymphocytic interstitial pneumonitis.

The different and only partially overlapping physiological roles and locations of IgG, IgA, and IgM suggest that there are several defects in the defense of the airways, only one of which is addressed by current IGRT. This helps to explain why patients with PAD experience recurrent respiratory tract infections even with regular IGRT. The most common infections are sinusitis and upper respiratory tract infections, but the range is much broader and not limited to the lungs (Figure 2).

Encapsulated bacteria such as *Haemophilus influenzae, Streptococcus pneumonia*, and *Moraxella catarrhalis* are the most common causative agents of recurrent infections. However, non-encapsulated, non-typeable strains of these bacteria have been identified as an important cause of pneumonia, sinusitis, bronchitis, and otitis in this patient population (30). Rhinovirus is another frequent causative agent of recurrent infections that leads to prolonged airway infection and inflammation in patients with hypogammaglobulinemia (31). Viral infection may render the airway mucosa susceptible for new bacterial infection (32) or aggravate bacterial infection as shown in patients with CF (33). In chronic obstructive pulmonary disease (COPD), rhinoviral infection exacerbates the existing microbiome with outgrowth of particular bacteria (34). *P. aeruginosa* should always be sought particularly where there is an evidence of structural damage (26). Also, *Mycoplasma* spp infections seem to play a role in pulmonary and non-pulmonary disease in patients with PAD (35–38).

### Subclinical Infections

Subclinical infections are well documented in patients with PAD, and a number of bacteria and viruses can be identified even in the periods when patients have no evident active infection. For example, a study of patient bronchoalveolar lavage fluid (BALF) detected bacteria in the lower respiratory tract of 9/14 asymptomatic patients, 6 of whom harbored erythromycin-resistant strains of *H. influenzae* (26). Adenoviruses were found in 4/14 patients; 2 of these patients had a dual infection with both adenovirus and rhinovirus (26). Similarly, a 12-month follow up study in 12 patients with PAD demonstrated the presence of respiratory viruses, most commonly rhinovirus, in the sputum in half of the 65 acute infection episodes (27). Moreover, virus elimination was much longer in patients with PAD than in healthy individuals. Rhinovirus shedding in patients with PAD lasted on average 40.9 days (95% CI: 26.4–55.4 days) compared to 11.4 (8.2–14.7) days and 10.1 (7.4–12.9) days in immunocompetent children and adults, respectively (27, 31). Not only did respiratory tract symptoms persist for the duration of virus shedding, but also new infections by another rhinovirus type appeared often soon after the first episode (31).

**Figure 3:**
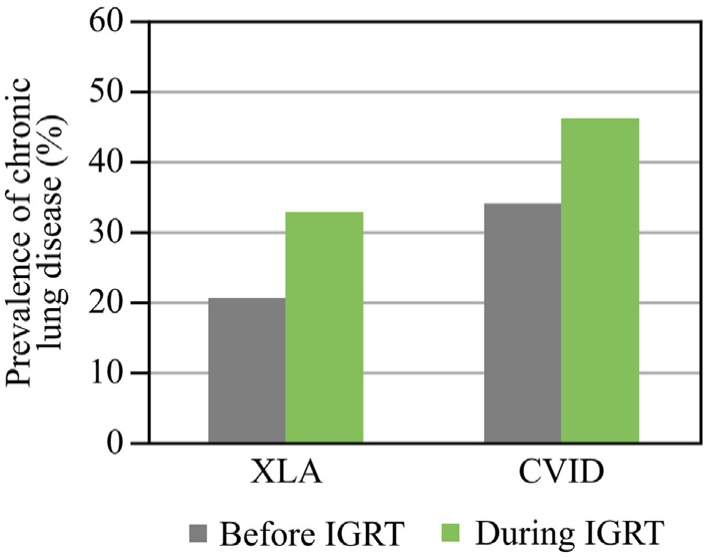
Prevalence of chronic lung disease before and during immunoglobulin replacement therapy (IGRT). Prevalence before and during IGRT is shown. Data for chronic lung disease are from Plebani et al. (XLA) (39) and Quinti et al. (CVID) (40). Abbreviations: CVID, common variable immunodeficiency; XLA, X-linked agammaglobulinemia.

In the long term, recurrent infections often lead to lung damage and chronic lung disease, with bronchiectasis being the most common complication. Studies with long follow up (up to 11 years) have shown that a substantial proportion of patients present with chronic lung disease at the time of diagnosis or develop it despite IGRT maintaining IgG levels to within the normal range (24, 39, 40) (Figure 3). The cumulative risk of chronic lung disease increases with disease duration and is not dependent on the age at diagnosis (39, 40). The rate of lung decline in PAD is much faster than that predicted in healthy individuals: the average decline of forced expiratory volume in 1 s (FEV1) in patients with CVID or XLA [45 mL/year (9)] is not only more than twofold higher than the normal age-related decline in healthy non-smoking individuals [19.6 and 17.6 mL/year for males and females, respectively (10)], but also higher than the decline in continuous smokers [38.2 and 23.9 mL/year in males and females, respectively (10)]. Even in one IVIG cycle, infections appear more frequently at the end of the cycle when trough Ig levels are low. A recent study showed that the risk ratio of a new infection was higher in the last compared to the first week of the IVIG cycle (1.55 for a 4-week cycle; *P* = 0.0314) (41). Therefore, it is likely that the optimization of trough IgG levels by increasing the total dose or shortening the interval will limit this difference in risk of infection. The stable IgG levels achieved using SCIG would also be expected to limit this in-dosing cycle variation.

### Bronchiectasis

**Figure 4:**
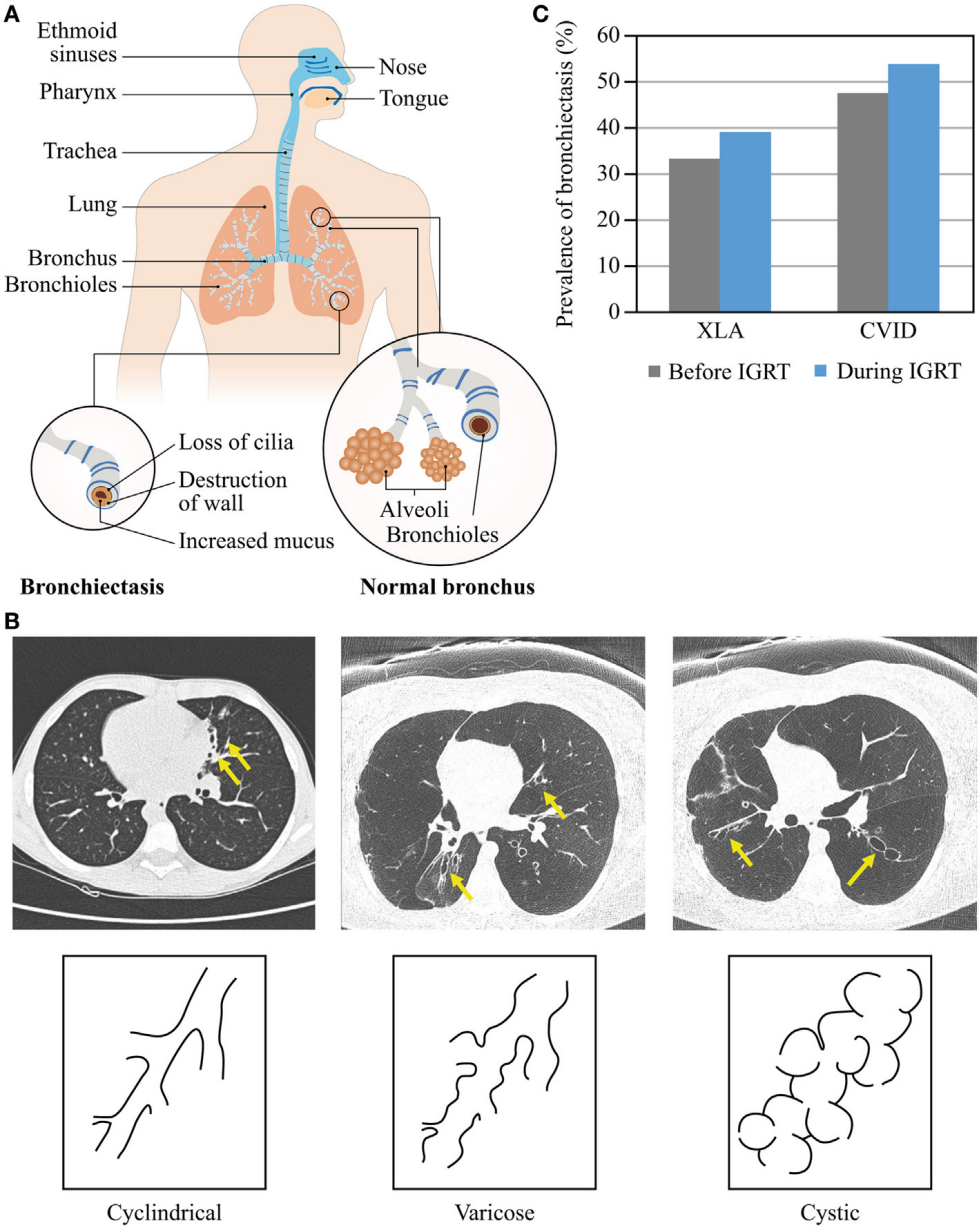
Morphology and prevalence of bronchiectasis. **(A)** Schematic presentation of normal bronchi and bronchi with bronchiectasis. The upper airways (blue) are more exposed to pathogens compared with the lower airways (orange). **(B)** Types of bronchiectasis. **(C)** Prevalence of bronchiectasis before and during IGRT. Data are from Quinti et al. (24).

Bronchiectasis is the irreversible scarring of the lung tissue resulting from infection and the ensuing inflammatory response (Figure 4A). Inflammation leads to excess mucus production and impaired mucociliary clearance, the latter in turn contributes to the increased number of bacteria residing in the lung, leading to a vicious cycle of inflammation and infection (42, 43). If inflammation lasts long enough or is severe enough, it leads to irreversible ulceration of the tissue with the involvement of fibroblasts and to scaring (44). Similar to other lung complications, bronchiectasis may develop despite regular IGRT (40). Morphologically, there are three types of bronchiectasis that reflect increasing severity of bronchial disease and its progression to the more central airways: cylindrical (also called tubular), varicose, and cystic (45, 46) (Figure 4B). The key symptoms of bronchiectasis are prolonged cough, excessive production of sputum, shortness of breath and wheezing, and chest pain. Severe and/or widespread bronchiectasis can lead to respiratory failure, atelectasis, and heart failure. Diagnosis of bronchiectasis is not straightforward and requires chest tomography imaging by either computed tomography (CT) or magnetic resonance tomography. Conventional chest X-ray imaging detects only a third of bronchiectatic lesions (47).

The prevalence of bronchiectasis among patients with PAD rises with age regardless of IGRT (Figure 4C). Over a 5-year observation period, the prevalence of bronchiectasis in patients with CVID increased from 47.3 to 53.7%, with age and serum IgA levels lower than 0.07 g/L being independent risk factors (24). Also in patients with XLA, the prevalence of bronchiectasis increased from 33 to 39% (24). The prevalence of chronic sinusitis increased with age in CVID and XLA patients, too, with low IgA serum levels being an independent risk factor in CVID (24, 39, 40). Moreover, because prevalence of chronic sinusitis was higher than prevalence of bronchiectasis in both patient cohorts, it is likely that bronchiectasis may develop late in the course of a common airway disease in PAD as a result of a chronic bacterial infection commencing in the upper airways, or as a sequel of prolonged inflammation, or both. Etiology of bronchiectasis may differ between CVID patients, as suggested by an increasing number of conditions with immune dysregulation rather than immunodeficiency. According to our own preliminary data in a large cohort of pediatric and adult CVID patients, IGRT does not appear to ameliorate the course of lung disease; the prevalence of bronchiectasis rises no less after establishing IGRT than with duration of disease.

Because the scarring of the lung tissue is irreversible, treatment of bronchiectasis focuses on preventing further tissue damage and alleviating the consequences. Treating the primary cause of bronchiectasis and reducing the risk of recurrent infections by optimization of IGRT and use of prophylactic antibiotics to stop the vicious cycle of inflammation are the primary goals. In addition, medication helping bronchodilation, mucus thinning, and expectoration can provide relief.

### New Therapeutic Approaches

New preventive and therapeutic approaches are urgently needed to alleviate lung damage in patients with PAD. Several potential treatments, including oral or inhaled antibiotics, inhaled hyperosmolar agents, mucolytic agents, airway clearance techniques, nasal irrigation, or nasal drug deposition have been tested in various settings associated with bronchiectasis (48). However, there are yet no published clinical trial results documenting their efficacy and safety in PAD.

Nasal irrigation and drug deposition in the nose or sinuses is a logical choice for delivering medication in patients with PAD similarly to immunocompetent individuals. The paranasal sinuses are poorly ventilated and often harbor chronic infections as in chronic sinusitis. Improving the sinus clearance may reduce the risk of infection in the lower airways. However, despite the fact that aerosolized drugs have been used for a long time, efficient drug delivery to the posterior nose and paranasal sinuses is still a challenge, because most aerosolized drugs are filtered by their passage through the nose (49). Irrigation of the nose using large-volume squeeze bottles has been shown to be effective (50), whereas nasal pump sprays could not deliver the aerosol to the sinuses (51). Pulsating aerosol delivery systems use a pulsating airflow to ventilate the sinuses and introduce the aerosolized medication (e.g., antibiotics and mucolytic agents) into the paranasal sinuses, which otherwise would be inaccessible (49). Studies using a paranasal nebulizer have shown that up to 6.5% of the nasally deposited drug was found in the sinuses (51). In addition, compared with nasal pump sprays, retention kinetics of the drug deposit in the nose was prolonged threefold (49, 52). This method can be used to deliver topical antibiotics, hyperosmolar agents, or even Ig directly in the airways or the lungs (53). In CF, application of tobramycin and recombinant human deoxyribonuclease (DNAse, Dornase alfa, Genentech, Inc., San Francisco, CA, USA) to the paranasal sinuses by oscillating pulse wave nebulization has proven beneficial for reduction of bacterial load and clinical symptoms (54, 55).

Antiviral treatment may be beneficial in patients who suffer from recurrent viral respiratory infections despite adequate IGRT. Preliminary studies in patients with PAD suggest that antiviral agents, such as nebulized ribavirin and pegylated interferon can reduce replication of rhinovirus at the airway site and improve symptoms (56). Patients with PAD do not appear to be prone to all viruses equally. Respiratory syncytial virus, which affects virtually all infants before they mount a specific immune response, does not appear to be a frequent pathogen in patients with PAD beyond infancy (57); this argues that IGRT affords protection against this respiratory pathogen. It is surprising that influenza infection, which regularly causes epidemics in the healthy population, has not received major interest in studies of PAD (57). Passive immunization by IGRT is likely to afford only limited protection against influenza, for donors (whose blood has been used to manufacture Ig products) have not been immunized against the most recent influenza strains. Moreover, active immunization of CVID patients induces only a weak, if any, antibody response (58). However, T-cell mediated immunity may respond better to influenza vaccination, supporting the common recommendation to immunize all patients with PAD (59). Recently, the first case of melanoma differentiation-associated protein 5 deficiency was reported in a patient with PAD; this inborn error resulted in susceptibility specifically to rhinovirus infection (60). Antiviral treatment might be useful in patients who suffer from recurrent viral respiratory infections despite adequate IGRT. Unfortunately, antiviral agents are not routinely available to treat all viral infections, including rhinovirus infection, and even if any were available there might be a risk of resistance with repeated or prolonged use.

In the past several years, research of the immune mechanisms controlling the susceptibility to chronic bacterial infection focused increasingly on the lung microbiota as a major factor informing and calibrating the immune system. The topic of the interaction of the microbiota with the host immune system is outside the scope of this work, and there are several excellent reviews summarizing the research to date (61–63). The “disappearing microbiota hypothesis” proposed by Blaser and Falkow offers an explanation for the increased burden of inflammatory disease as a result of changing hygiene, human macroecology, and clinical practice (64). The lung is a complex microbial ecosystem, in which different pathogens compete for survival not only with one another but also with the commensal microbiota (62). Animal studies have shown that not all members of the microbiota have an equal ability to influence the immune system, thus the disruption of the microbiota composition may result in a reduced host defense against bacterial and viral infection (65–67). Also, studies have shown that microbiota-depleted mice had significant loads of bacteria and viruses compared with mice with normal microbiota. In humans, the lung microbiota differs between health and inflammation, whereby in the latter situation the broad range of microbiota is substantially reduced, appearing “missing” or “simplified” (62, 64, 68). For example, subjects with asthma or COPD have a distinctive lung microbiota composition compared to healthy controls (69, 70). In patients with CVID, inflammatory complications are associated with reduced within-individual bacterial diversity, and dysbiosis (i.e., an unhealthy imbalance in the normal bacterial ecology), and elevated immune cell activation markers in the gut (71). Evaluating the pulmonary microbiome may evolve as a new diagnostic tool to assess the risk of progression of lung disease. Active modification of the pulmonary microbiome in patients with PAD may reduce ecological niches for pathogens and have a beneficial effect on host defense even with an immune system that functions only in part. Although research of the lung microbiota lags substantially behind that of the gut microbiota, modifying (or restoring) the microbiota of the airway mucosa using probiotics, similar to the approach used in the gut, may help in maintaining a favorable immunological balance (63).

### Adjunctive Therapies

The recently completed first study on antibiotic prophylaxis in Italian patients with PAD evaluated the efficacy and safety of low doses of azithromycin (250 mg three times per week on three consecutive days) for 24 months vs placebo (72). The results of this study will provide much needed evidence of the efficacy of antibiotic prophylaxis. Regarding the practice of antibiotic usage for the treatment of respiratory infections, a recent prospective study reported that patients delay antibiotic treatment by a median of 5 days until they suffer specific “warning” symptoms, such as cough, shortness of breath, and purulent sputum (73). As the authors of this article point out, this finding is unexpected, as patients should have access to antibiotics for immediate use and are advised to start treatment when they have a breakthrough infection. Whether this delay is a matter of patient choice or of access to healthcare remains to be investigated. In addition, only exacerbations characterized by purulent sputum appeared to respond to use of antibiotics, while exacerbations with signs of upper respiratory tract infections did not. Moreover, exacerbations were as frequent in patients using prophylactic antibiotics, as in those using antibiotics on demand. It remains unclear whether the outcomes would have been different if antibiotic treatment was commenced earlier than 5 days after onset of symptoms. Finally, respiratory viruses were detected more frequently than bacterial pathogens. These findings argue for a careful evaluation of the causing pathogen as guidance for the use of antibiotics.

Hyperosmolar agents, most commonly hypertonic saline, have been used to break the vicious cycle of mucus retention and infection in patients with bronchiectasis (74). Another approach to improve mucus elimination is using mucolytic agents (carbocysteine orally or nebulized DNAse) to increase mucus fluidity and facilitate expectoration and, while this approach has been used in selected PAD patients, there are no controlled studies in PAD. The observation that DNAse improves lung function parameters (FEV1) in patients with CF (75), while it leads to FEV1 deterioration in patients with non-CF bronchiectasis (76), indicates that the efficacy of therapeutic interventions may differ substantially between various pulmonary conditions. Therapeutic strategies, therefore, need to be carefully assessed in controlled clinical trials in patients with PAD.

## Non-Infectious Pulmonary Complication in PAD

Non-infectious pulmonary complications in PAD are common and contribute substantially to morbidity. Thus, in CVID, two independent studies have shown that patients with disease-related non-infectious complications have a significantly poorer survival prognosis than those without complications: the risk of death was estimated to be 11-fold higher than that in patients with infections only (*P* < 0.001) (7, 8). Approximately 30% of patients with CVID have diffuse interstitial lung disease (ILD) (51). Etiology is unknown in most cases, but multiple monogenic causes have been identified (77).

Non-infectious pulmonary complications include different forms of ILD, such as granulomatous-lymphocytic ILD (GLILD), cryptogenic organizing pneumonia (COP), lymphocytic interstitial pneumonitis (LIP), follicular bronchiolitis (FB), and/or lymphoid hyperplasia. Screening with high-resolution computed tomography (HRCT) chest scan indicates a much higher percentage of patients with ILD and bronchiectasis among those with PAD than previously appreciated (78). Establishing a correct pathological diagnosis is crucial for treatment of ILD. For instance, COP is responsive to steroids, whereas in GLILD, other treatments may be needed.

### Granulomatous-Lymphocytic ILD

Granulomatous-lymphocytic ILD is the pulmonary component of a non-necrotizing, systemic disease characterized by adenopathy, splenomegaly, and granulomatous inflammation that may affect not only the lung, but also the liver, bone marrow, and lymph nodes (79, 80). The prevalence of GLILD in patients with PAD is unknown; however, it is found in CVID and an increasing number of monogenic disorders. Approximately 20% of patients with GLILD present with polyclonal lymphocytic infiltration or nonmalignant hyperplasia of the lymph nodes in addition to granuloma (80). GLILD typically occurs in the context of CVID; so far, GLILD has not been described in congenital agammaglobulinemia.

**Figure 5:**
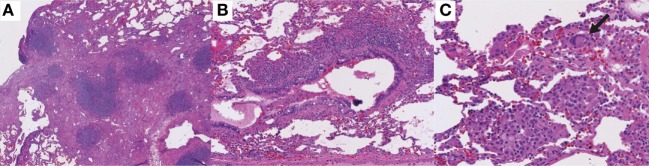
Histologic features of granulomatous-lymphocytic interstitial lung disease. **(A)** Lymphocytic interstitial pneumonitis. Diffuse, lymphocytic interstitial infiltrates obliterating the alveolar spaces in some areas (100×). **(B)** Follicular bronchiolitis. Nodular, lymphoid peribronchiolar aggregates (100×). **(C)** Non-necrotizing granuloma with multinucleated giant cells (arrow; 200×). Hematoxylin and eosin staining.

The key histopathological features of GLILD are LIP, FB, and non-necrotizing granuloma (Table 1; Figure 5). These histopathological features are numerically more frequent in the lower lung zones and are found in the same biopsy in nearly all patients. LIP often manifests as a mix of moderate to severe peribronchiolar and interstitial lymphoid infiltration. The predominant cells in the infiltrate are CD4+ T cells, but nodules of CD20+ B cells surrounded by CD4+ T cells are also found, predominantly localized to the interstitium (81). Distinct B-cell follicles and T-cell areas have been observed also in patients with CVID and lymphoid hyperplasia (82). Surprisingly, regulatory T cells are absent in the lungs in GLILD (81). This latter observation is in agreement with previous reports indicating reduced numbers and function of regulatory T cells in blood in CVID associated with GLILD (83–86). Granulomas are non-necrotizing, poorly to well-formed, and widely distributed, but with lower lung zone predominance, occasionally in association with interstitial inflammation (81).

**Table 1:** Histopathological features of GLILD.

Feature^a^	Occurrence (*N* = 16)	Severity		Key features
Lymphocytic interstitial pneumonitis	100%	Mild	19%	Lymphocytic infiltration of variable density—peribronchiolar and interstitial
		Moderate	25%	
		Severe	56%	
Follicular bronchiolitis	100%	Mild	25%	Consistently present; nodular peribronchial inflammation associated with interstitial inflammation
		Moderate	56%	
		Severe	19%	
Non-necrotizing granuloma	93.75% (15/16 cases)	Mild	50%	Well, moderate, or poorly circumscribed; non-necrotizing, may be cuffed by lymphocytes or associated with lymphoid infiltration; random distribution
		Moderate	31.25%	
		Severe	12.5%	
Organizing pneumonia	87.5% (14/16 cases)	Mild	43.75%	Variable severity with Masson bodies and other typical features of COP
		Moderate	37.5%	
		Severe	6.25%	
Interstitial fibrosis	75% (12/16 cases)	Mild	31.25%	Variable severity from patchy to extensive areas of collagenized fibrosis
		Moderate	25%	
		Severe	18.75%	

*^a^Data from Rao et al. (81)*.

*COP, cryptogenic organizing pneumonia; GLILD, granulomatous-lymphocytic interstitial lung disease*.

In addition to LIP, FB, and granuloma, organizing pneumonia and interstitial fibrosis are also found in most patients with GLILD and are moderate or severe in approximately half of the cases (81). Organizing pneumonia is characterized by Masson bodies amidst pale, myxoid stroma within the alveoli and adjacent interstitium. Interstitial fibrosis is characterized by areas of collagenized fibrosis, with the more severe cases being accompanied by alveolar remodeling (81). Whether GLILD causes progressive pulmonary fibrosis is somewhat controversial. However, moderate to severe pulmonary fibrosis has been found in 44% of patients with GLILD strongly suggesting this leads to progressive pulmonary fibrosis (78, 87, 88). However, the presence of moderate to severe interstitial fibrosis in the lung biopsies of nearly 50% of patients with GLILD strongly argues this is a progressive lung disease in most patients.

**Figure 6:**
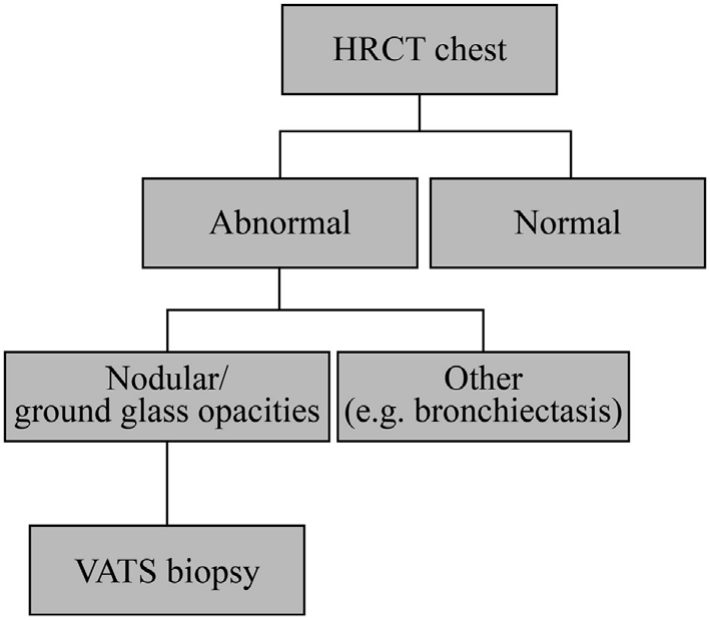
Granulomatous-lymphocytic interstitial lung disease diagnostic algorithm. All patients with common variable immunodeficiency should have a high-resolution computed tomography (HRCT) of the chest to screen for pulmonary disease. If there is significant interstitial lung disease (ground glass abnormalities, nodules, and areas of consolidation), one should consider lung biopsy by video-assisted thoracoscopic surgery (VATS) for definitive diagnosis. It may be worth considering if a more easily accessible organ such as a lymph node could be useful for detection of granulomas.

**Figure 7:**
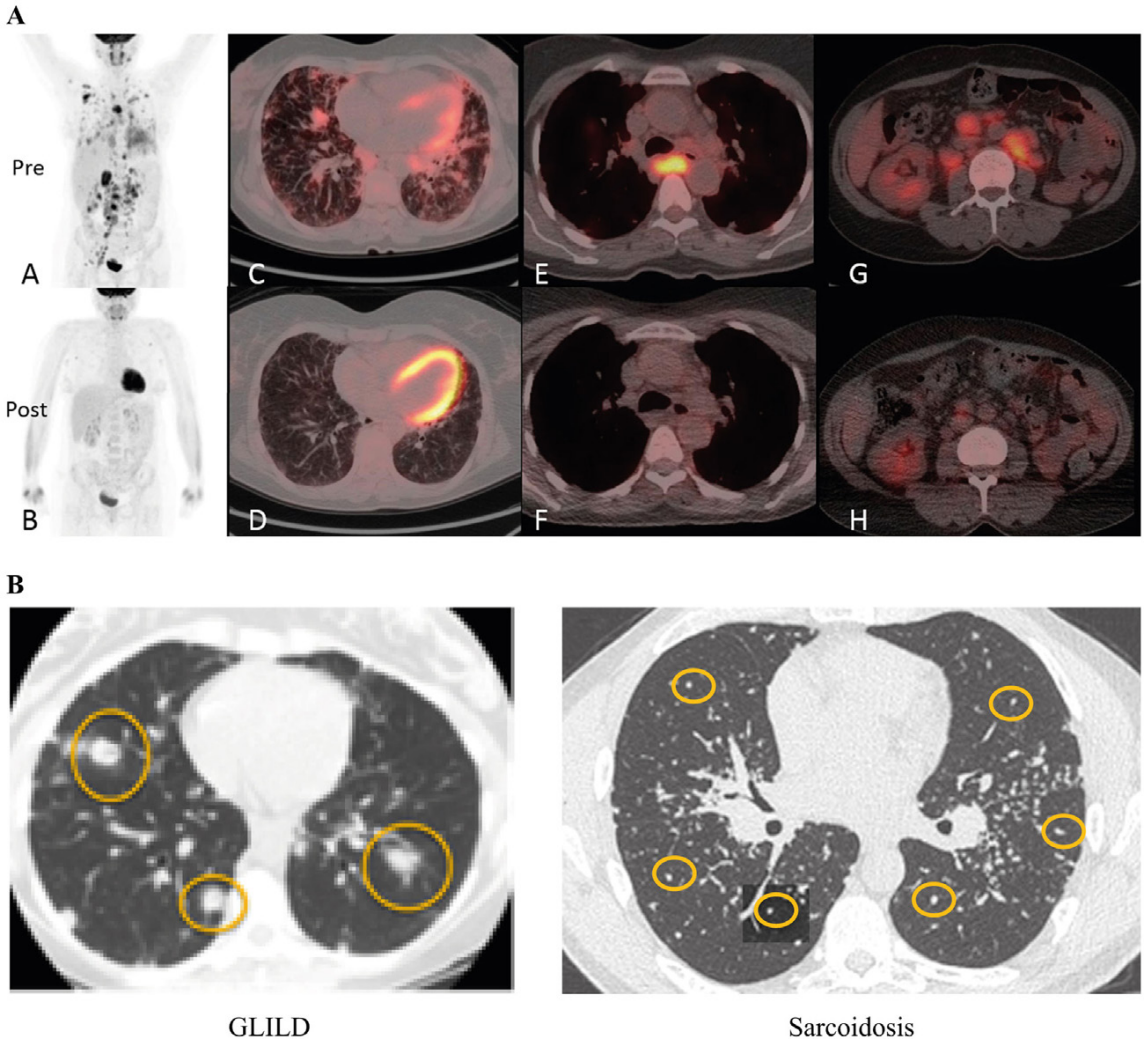
Diagnosis of granulomatous-lymphocytic interstitial lung disease (GLILD). **(A)** PET-CT before and 3 months after rituximab and mycophenolate treatment. Whole body images were acquired at 90 min following 285 MBq 2-[(18)F]-fluoro-2-deoxy-d-glucose (tracer). Maximum intensity projection whole body images before treatment show widespread abnormal uptake of tracer in the lung parenchyma and lymph nodes above and below the diaphragm (A); images following treatment show near resolution of all the areas of abnormal tracer uptake (B). (C,D) Axial fused positron emission tomography–computed tomography (PET–CT) images at the mid-thoracic level. The level of tracer uptake is represented by the intensity of color superimposed upon the CT image. There is a combination of interstitial septal thickening and ill-defined tracer-avid peri-bronchovascular nodules before treatment (C); post-treatment image shows improvement of the nodularity with near resolution of tracer uptake (D). Images at the level of the carina show an enlarged lymph node exhibiting intense abnormal tracer uptake prior to treatment (E), and a normalized lymph node with no abnormal tracer uptake following treatment (F). Pre-treatment images through the abdomen at the level of the right renal hilum demonstrate numerous enlarged tracer-avid retroperitoneal lymph nodes (G) which reduce in size and tracer uptake following treatment (H). Reproduced with kind permission from 2016 British Society for Immunology, Clinical and Experimental Immunology, 187: 138–145 (100). **(B)** Radiographic features of GLILD versus sarcoidosis. GLILD is characterized by macronodular (yellow circles) disease that is located predominantly in the lower lung zones. In contrast, micronodular (yellow circles) disease located in the upper lung zones and frequently accompanied by marked hilar adenopathy (stars) is characteristic of sarcoidosis. Reproduced from Ref. (101) with kind permission from European Respiratory Society.

Suspicion for GLILD should be raised particularly with the presence of lymphadenopathy, splenomegaly, autoimmunity, or a low number of class switched memory B-cells. Diagnosis of GLILD is based on HRCT of the chest (Figure 6) and, possibly, lung biopsy. Typical radiographic features of GLILD include large nodules and small nodules, areas of consolidation, and ground glass abnormality predominantly located in the lower lung zone, and frequent mediastinal adenopathy. The differential diagnosis of diffuse parenchymal disease in patients with CVID is extensive and includes LIP, nonspecific interstitial pneumonia, usual interstitial pneumonia, sarcoidosis, hypersensitivity pneumonitis, COP, low- and high-grade lymphoma, GLILD, as well as infection. To make a definitive diagnosis in a patient with suspected GLILD, we (JR) recommend a biopsy *via* video-assisted thoracoscopic surgery (VATS) with sampling of at least two lobes of the lung. Several studies have demonstrated that VATS is superior to transbronchial biopsy in determining a definite diagnosis in cases of suspected ILD (89–94). As previously mentioned, most patients with GLILD have significant areas of organizing pneumonia. Therefore, a transbronchial biopsy may lead to the misdiagnosis of COP due to sampling artifact. Similarly, transbronchial biopsy may yield insufficient tissue to make the diagnosis of lymphoma, particularly low-grade lymphoma. VATS also provides additional prognostic (extent of fibrosis) and diagnostic (e.g., B-cell lymphoma of low or high grade) information that may not be obtained by transbronchial biopsy. The 30-day post-operative mortality rate is 0–6% (89–93, 95, 96), which varies according to the clinical status of the patient as well as the expertise present in a given center (91, 92, 95, 97). Complications include pneumothorax, pulmonary atelectasis, lower respiratory tract infections, poor lung expansion, and more rarely (<5%) surgical emphysema, prolonged neuropathic pain, delayed wound healing, persistent air leak, acute respiratory distress, and hemothorax (91, 92, 95, 97). In children, VATS is recommended only if the diagnosis is uncertain despite thorough clinical evaluation or therapeutic decisions cannot be made without histology (98, 99). Positron emission tomography (PET) with 2-[(18)F]-fluoro-2-deoxy-d-glucose (FDG) combined with CT allows identification of active lymphoproliferative sites early in the inflammatory process and shows the systemic nature of the condition (Figure 7A) (100). In addition, FDG PET-CT shows sites of lymphoproliferation that may be more amenable for biopsy and confirmation of granulomatous inflammation than the lung. An additional alternative approach for confirming granulomatous histology would be using the tissue obtained at splenectomy if this had been undertaken. Obtaining tissue from more accessible organs should also be considered. For example, a skin biopsy can help to confirm or exclude sarcoidosis.

While there is no universally accepted diagnostic process for GLILD, decisions are made individually and further studies will be needed to define the optimal approach.

Differentiating between GLILD and sarcoidosis can be potentially challenging, owing to the systemic nature of both disorders and the somewhat similar pathological findings (80). While both sarcoidosis and GLILD have non-necrotizing granuloma, FB and LIP are much more prominent in GLILD. Radiographically, the abnormalities in sarcoidosis are predominantly in the upper lung zones and consist of micronodular disease and a pronounced hilar adenopathy, whereas GLILD is characterized by micronodular and macronodular disease, diffuse adenopathy with less prominent hilar adenopathy, and lower lung zone predominance (Figure 7B). Bronchiectasis is uncommon in sarcoidosis, but occurs in 20–50% of patients with GLILD. In addition, plasma cells are present in the lung in sarcoidosis, but not in GLILD, consistent with the general absence of plasma cells in GLILD and CVID.

Monogenic disorders causing CVID-like diseases have now been well described in patients with GLILD: mutations in the transmembrane activator and calcium-modulator and cyclophilin ligand interactor (TACI), cytotoxic T-lymphocyte-associated protein 4 (CTLA-4) haploinsufficiency (102), lipopolysaccharide responsive beige-like anchor protein (LRBA) deficiency (103), mutations in the X-linked inhibitor of apoptosis protein (XIAP), hypomorphic recombinase-activating gene 1 (RAG1) mutations (104, 105), and lysine methyltransferase 2D (KMT2D) mutations (106). Patients with enteropathy, immune cytopenias, or other autoimmune or central nervous system diseases should raise the suspicion of LRBA deficiency or CTLA4 haploinsufficiency. Overall, 66% of patients with CTLA-4 deficiency were found to have GLILD (107, 108). LRBA functions to maintain the intracellular stores of CTLA-4 (109), and, therefore, LRBA deficiency and CTLA-4 deficiency have a similar clinical phenotype. The question whether all CVID patients should undergo at least targeted DNA sequencing remains to be answered (110–112). Determining the genetic cause of CVID might result in a change of treatment of various complications and improve prognosis. However, patients with a severe inflammatory/autoimmune disease, early presentation (younger than 10 years), multiple affected family members or very low numbers of B cells may have a higher likelihood of identification of a causative gene using next-generation sequencing approaches (112).

There are no guidelines and few reports on treatment of GLILD. However, the presence of both B and T cells in the infiltrate suggests a combination immunosuppressive therapy targeting both cell populations might be beneficial. Indeed, a combination of rituximab and azathioprine improved radiographic abnormalities and pulmonary functional tests (PFTs) in seven patients with CVID and GLILD (113). Rituximab (375 mg/m^2^/week) is administered intravenously once a week for 4 weeks, and this course is repeated 3–4 times at 4–6-month intervals. Oral azathioprine is given concomitantly for 18 months. In patients with poor tolerability for azathioprine, its active metabolite, 6-mercaptopurine, can be used. If the patient does not tolerate either of these drugs, mycophenolate mofetil is also effective. The dosage of these immunosuppressants needs to be adjusted to maintain an adequate lymphocyte and neutrophil count. Other immunosuppressive medications that have been reported to have a beneficial effect in GLILD include abatacept in patients with LRBA or CTLA4 haploinsufficiency (109, 114), tumor necrosis factor-α inhibitors (115), and corticosteroids (116). In our experience, corticosteroids do not lead to a durable remission. A double-blind placebo-controlled trial is needed to define the natural history of GLILD and document efficacy of immunosuppressive approaches in GLILD.

## Assessment of the Lung in PAD

### Pulmonary Function Tests

Pulmonary function tests provide useful information on lung performance and on the extent and progression of chronic lung disease in patients with PAD, in addition to the overall clinical assessment and physical examination. The most commonly performed PFT is spirometry providing data on the forced vital capacity (FVC) and FEV1: abnormal FVC and FEV1 readings may indicate restrictive and obstructive lung disease, respectively. In patients with PAD, decline in FEV1 has been shown to be a more sensitive indicator of respiratory function than FVC, as expected due to the obstructive nature of bronchiectasis (117). In addition to assessments performed by spirometry, regular assessment of diffusing capacity for carbon monoxide (DLCO) in suspected ILD is useful alone (117). This is one of the reasons why care of CVID patients should be managed by specialized centers that have access to other specialties, including a pulmonology liaison service. DLCO should also be evaluated in all patients with splenomegaly and/or lymphadenopathy.

In a study of 37 patients with CVID and XLA, the average annual decline in FEV1 was 45 ± 6 mL/year (9), which exceeds the normal rate of decline of 19.6 and 17.6 mL/year in healthy non-smoking males and females, respectively (10). In this study, lung function decline correlated inversely with IVIG dose (*P* = 0.004), indicating that higher IVIG doses are associated with preservation of lung function, although the correlation between trough IgG levels and FEV1 decline was not statistically significant (*P* = 0.485). In another study of 20 patients with PAD, a clear correlation between higher IgG trough levels and preservation of FEV1 (*R*^2^ = 0.2688; *P* = 0.0281) was observed (117). A significant correlation between serum IgG trough level and FVC and FEV1 values was found also in 12 patients with hypogammaglobulinemia and ILD and led to the conclusion that the minimal target serum IgG level should be 5 g/L (118). Collectively, these studies suggest higher doses of IgG may be considered for patients showing deteriorating lung function; however, maximum recommended trough IgG levels have not been determined. The decline in FEV1 also appeared slower in patients who spent longer periods of time on antibiotics; however, the correlation was not significant (*P* = 0.1195) (117).

Based on these rates of decline in FEV1, we recommend at least annual PFT monitoring (including DLCO if available) for patients with PAD older than 5 years of age (117). Testing more frequently may be appropriate for those experiencing a more rapid decline of lung function (119).

Recently, several clinical trials employed a new technology, the multiple breath washout technique (MBW). MBW assesses the quality of ventilation, indicated by the lung clearance index (LCI) during regular breathing. This method is not only less dependent on the cooperation of the patient, thus allowing use in children as young as age 4, but also appears more sensitive to minor changes at an early stage of lung disease and more meaningful in advanced disease, as shown in CF (120), primary ciliary dyskinesia (121), and non-CF bronchiectasis (122), in all of which LCI correlated better to the extent of bronchiectasis than FEV1. Finally, MBW appeared also more sensitive for assessing changes in lung disease in clinical trials with relatively short-term interventions. Inhalation of hypertonic saline in a small group (*n* = 20) of children with CF over a period of 4 weeks resulted in a significant improvement of LCI (treatment effect 1.16 ± 0.94), but not of spirometry parameters (123). However, to observe a significant change in FEV1 [% predicted] with this intervention, a sample size of 351 patients would have been required.

### High-Resolution CT

Pulmonary functional tests have a low sensitivity to screen for pulmonary complications such as ILD and bronchiectasis (124) and, therefore, should be combined with imaging techniques. The sensitivity of HRCT for detection of pulmonary complications in patients with CVID is superior to both chest X-ray and PFTs (125), but the method is associated with higher costs and radiation doses. Radiation dose is a potential concern for patients with CVID, who in some cases may be more radiosensitive than healthy subjects (126–128), particularly where over time repeated imaging may be clinically necessary. For these patients, and especially for children, CT techniques that use lower radiation doses should be considered. Although low-dose computed tomography (LDCT) uses less ionizing radiation than conventional CT scans, the predictive effective doses used in LDCT of the lungs may still be 4–12 times greater than those used in conventional X-rays (129). A possibility to further reduce the radiation dose in lung CT scans would be to use the model-based iterative reconstruction (MBIR) technique for ultra-low-dose chest CT. MBIR provides an almost 80% reduction in radiation dose for chest CT without reducing sensitivity (130).

HCRT can be critical in the early diagnosis of airway abnormalities (airway disease) and ILD, which cannot be differentiated by PFTs (131). In patients with CVID, HRCT revealed airway disease in 20% of pediatric patients and 30% of adults, and ILD in 15% of children and 34% of adults, respectively (124, 131). In the adult population, the presence of ILD correlated positively with autoimmune disease and markedly lower CD4+ T cell, naïve CD4+ T cell, and naïve and switched memory B-cell counts (124). Both airway disease and ILD are treatable diseases but differ in etiology and require different treatment. While high-dose IGRT may impact progression of airway disease in patients with CVID, ILD responds to immunosuppressive therapy. In pediatric CVID patients with ILD, nodules on HCRT disappeared following treatment with steroids, suggesting that early ILD may be reversible (131). As ILD appears to be asymptomatic during the initial stage, screening of all patients with CVID should facilitate early disease detection and enable early therapy which could be less aggressive (131).

There is a need for standardized HCRT scoring methods to facilitate efficient screening and follow-up for pulmonary complications associated with CVID (131, 132). To aid the assessment of unrecognized lung disease progression in these patients, the scoring system should be based on abnormalities specific for PAD, rather than adapted from other diseases such as CF. A scoring method was developed in a cohort of 54 children with stable CVID or CVID-like disorders (132). In this pediatric study population, lung abnormalities were common (found in 85–95% of patients), although the overall extent and severity of abnormalities were mild. Bronchial pathology was the most common pathology, observed in up to half and one-third of the patients with bronchial wall thickening and bronchiectasis, respectively. However, interstitial pathology and ventilation pathology were described as affecting a third (air trapping) and a quarter (nodules) of the patients. The latter was effectively treated with corticosteroids in some cases. The Chest CT in Antibody Deficiency Group is currently developing a quantitative scoring system that can be used as a validated tool in multicenter studies and could help to endorse uniform documentation of chest CT scans (133).

### Magnetic Resonance Imaging (MRI) With Diffusion Weighted Imaging (DWI)

Magnetic resonance imaging with DWI offers a radiation-free alternative to the HRCT scan. MRI has been shown to be non-inferior to HRCT in identifying bronchial and parenchymal abnormalities, although HCRT had a higher capacity in identifying peripheral airway abnormalities (134). PFT alterations correlated well with MRI bronchial abnormalities, but not with MRI parenchymal scores (134). The implementation of DWI facilitates the acquisition of information about the microstructure of the tissue and may enable detection of areas of inflammation and subclinical infection in lung parenchyma (134). However, although promising, MRI DWI is technically demanding, requires an expert radiologist, and possibly necessitates sedation in children.

### Inflammatory and Clinical Biomarkers

To determine the need of anti-inflammatory drugs as adjunctive therapy in PAD, reliable biomarkers are required. Potentially useful biomarkers of infection risk are the presence of sinusitis, low IgG trough levels, low IgA, and bronchiectasis. Similarly, the following symptoms could be valuable as biomarkers of non-infectious inflammation: low class-switched memory B-cells, autoimmunity, low T cells and naïve T cells, splenomegaly, lymphadenopathy, and potentially elevated IgM and beta 2 microglobulin (100). However, data on biomarkers in peripheral blood are not available, except for low endogenous plasma IgG concentrations measured prior to IGRT and a deficiency of class-switched memory B cells (24, 135, 136). IgM levels are not always low in patients with CVID, but they were reported to be lower in patients experiencing recurrent infections compared with those with fewer infections (24). Splenomegaly can be a biomarker for GLILD, given the multi-systemic nature of the disease (81). Abnormalities in the T-cell population found in BALF, such as a low CD4/CD8 ratio, can be associated with poor functional outcomes including FEV1 and FVC (137). However, bronchoalveolar lavage for T-cell assessment is an invasive technique and thus it is usually used only in patients with severe disease. Other potential inflammatory markers in spontaneously expectorated (138) or induced (139) sputum, including cellular content, cytokines, reactive oxygen metabolites, and bacterial density (140) have been evaluated in various conditions including CVID, yet before they can become a reality in this setting, further studies are needed (43). Likewise, there are no reliable data of the usefulness of beta 2 microglobulin in patients with PAD.

Microbiological surveillance of patients with PAD is of great clinical importance. Although it is currently unclear how many episodes of bacterial infection in PAD are preceded by viral infections of the upper airway (2), appropriate microbiological surveillance may help understand and prevent infection progression. The surveillance protocol in patients with PAD may include regular chest HRCT and PFTs (including DLCO and possibly LCI) or MRI with DWI as an alternative to CT with optional sputum analysis and molecular detection with polymerase chain reaction (3, 4).

## Conclusion

Pulmonary morbidity in patients with PAD advances despite apparently adequate IGRT. New preventive and therapeutic approaches are urgently needed. Clinical trials to evaluate these approaches will rely on new ways of assessing and documenting lung disease. Optimal pulmonary management must encompass the upper airway, the “gateway to the lung.” Non-infectious complications such as non-infectious lymphoproliferation, where GLILD represents the pulmonary component, contribute substantially to morbidity in these patients, and requires specific treatment. Regular assessment of the structural and functional condition of the lung as well as an improved understanding of subclinical recurrent infections will, we hope, inform therapeutic decisions aiming to reduce lung damage.

## Key Take-Home Messages

- Lung disease, both infectious and non-infectious, progresses despite IGRT
- Characteristics making lung disease more likely include existing inflammatory/autoimmune disease, lymphadenopathy, splenomegaly, low counts of class-switched memory B cells, and CD4+ T cells
- Improved molecular characterization of gene defects has led to identification of several monogenic defects which appear to be associated with granulomatous-lymphocytic ILD (mutations in TACI, CTLA-4 haploinsufficiency, or LRBA deficiency, mutations in XIAP, hypomorphic RAG1 mutations, and KMT2D mutations)
- A better understanding of the natural history and pathogenesis of ILD in PAD is needed
- Early diagnosis may allow interventions to prevent or slow end organ damage
- New and improved PFT modalities now exist and warrant further study for utility in PAD
- Low-dose CT scans and radiation-free alternative scans offer an improved safety profile in PAD
- Standardized multi-center documentation of chest CT scans is one of the tools for providing better data on natural history and interventions
- Optimal pulmonary management of patients with PAD must encompass the upper airway, which serves as the “the gateway” to the lungs
- There are few clinical studies evaluating potential treatments of lung disease in PAD
- Early targeted intervention may reduce the burden of steroids currently employed, which are used over long periods of time with only partial efficacy
- New therapies are needed to relieve the burden of viral infections

## Author Contributions

All authors contributed to the same extent to the development of this article.

## Conflict of Interest Statement

UB has received honoraria, expenses, and consulting fees from Baxalta, now part of Shire, Biotest, CSL Behring, and Octapharma, and research grants from CSL Behring and Baxalta, now part of Shire. JR has received support from CSL Behring for participating in the CSL Behring sponsored satellite symposium at the ESID 2016 Annual Meeting. PS-P has received advisory board, speaker, or project support from Grifols; support from CSL Behring for attending the ESID 2016 Annual Meeting. SJ has received support for speaker, conference, advisory board, clinical trial, or projects from CSL Behring, Shire, Octapharma, Biotest, LFB, UCB Pharma, GlaxoSmithKline, Swedish Orphan Biovitrum, Binding Site, Grifols, Zarodex, and Weatherden, and support from LFB for attending the ESID 2016 Annual Meeting.

## Abbreviations

BALF, bronchoalveolar lavage fluid; CF, cystic fibrosis; CI, confidence interval; COP, cryptogenic organizing pneumonia; COPD, chronic obstructive pulmonary disease; CT, computed tomography; CTLA-4, cytotoxic T-lymphocyte-associated protein 4; CVID, common variable immunodeficiency; DLCO, diffusing capacity for carbon monoxide; DNase, recombinant human deoxyribonuclease (dornase alfa); DWI, diffusion weighted imaging; ESID, European Society for immunodeficiencies; FEV1, forced expiratory volume in 1 s; FVC, forced vital capacity; GLILD, granulomatous-lymphocytic interstitial lung disease; HRCT, high-resolution computed tomography; Ig, immunoglobulin; IGRT, immunoglobulin replacement therapy; ILD, interstitial lung disease; IVIG, intravenous immunoglobulin G; KMT2D, lysine methyltransferase 2D; LCI, lung clearance index; LDCT, low-dose computed tomography; LIP, lymphocytic interstitial pneumonitis; LRBA, lipopolysaccharide responsive beige-like anchor protein; MBIR, model-based iterative reconstruction; MBW, multiple breath washout technique; MDA5, melanoma differentiation-associated protein 5; MRI, magnetic resonance imaging; PAD, primary antibody deficiencies; PET, positron emission tomography; RAG1, recombinase-activating gene 1; TACI, transmembrane activator and calcium-modulator and cyclophilin ligand interactor; VATS, video-assisted thoracoscopic surgery; XIAP, X-linked inhibitor of apoptosis protein; XLA, X-linked agammaglobulinemia.
